# Access to primary care and cognitive impairment: results from a national community study of aging Americans

**DOI:** 10.1186/s12877-021-02545-8

**Published:** 2021-10-20

**Authors:** Megan A. Mullins, Julie P. W. Bynum, Suzanne E. Judd, Philippa J. Clarke

**Affiliations:** 1 Center for Improving Patient and Population Health and Rogel Cancer Center, University of Michigan, North Campus Research Complex, Bldg 16, Room 409E, 2800 Plymouth Road, Ann Arbor, MI 48109 USA; 2 Institute for Healthcare Policy and Innovation, University of Michigan, Ann Arbor, MI USA; 3 Division of Geriatric & Palliative Medicine, University of Michigan School of Medicine, Ann Arbor, MI USA; 4 Department of Biostatistics, University of Alabama at Birmingham, Birmingham, AL USA; 5 Department of Epidemiology and Institute for Social Research, University of Michigan, Ann Arbor, MI USA

**Keywords:** Primary care, Access to care, Cognitive impairment, Dementia

## Abstract

**Background:**

Despite a growing burden of Alzheimer’s Disease and related dementias (ADRD) in the US, the relationship between health care and cognitive impairment prevention is unclear. Primary care manages risk causing conditions and risk reducing behaviors for dementia, so we examine the association between individual and area-level access to primary care and cognitive impairment in the REasons for Geographic And Racial Differences in Stroke (REGARDS) study.

**Methods:**

REGARDS participants with a cognitive assessment and vascular measurements at their baseline visit were included in this cross-sectional analysis. Cognitive impairment was defined as a Six-Item Screener (SIS) score < 5. Primary care supply, primary care utilization and emergency department (ED) utilization were measured at the primary care service area (PCSA) level based on participant’s address. Individual access to care was self-reported. Models were adjusted for confounding by demographics, socioeconomic status and behavioral risk factors.

**Results:**

Among 25,563 adults, living in a PCSA with low primary care supply was associated with 25% higher odds of cognitive impairment (OR 1.25 CI 1.07-1.45). Not having a regular source of medical care was associated with 14% higher odds of cognitive impairment (OR 1.14 CI 1.02-1.28), and living in a PCSA with high emergency department utilization was associated with 12% higher odds of cognitive impairment (OR 1.12 CI 1.02-1.23).

**Conclusions:**

Our results are an important first step in understanding how health care may prevent cognitive impairment. They highlight the importance of primary care and suggest future work clarifying its role in preventing cognitive decline is imperative.

**Supplementary Information:**

The online version contains supplementary material available at 10.1186/s12877-021-02545-8.

## Background

As the American population ages, projections estimate that by 2050, one new case of Alzheimer’s disease will develop every 33 s for an additional 1 million new cases each year [1]. People with Alzheimer’s disease or related dementias (ADRD) use more health care, including extended hospitalizations, trips to the emergency department and home health care [2–4]. Although the healthcare costs of ADRD are well documented (largely attributed to the costs of hospitalization and long term care) [5], aspects of the healthcare environment that are relevant for preventing cognitive impairment are not well understood [6, 7].

Most existing ADRD primary care research has focused on the role of ambulatory/primary care for detecting cognitive impairment and dementia; however, little is known about the role of primary care in preventing cognitive decline [8]. Indeed, little research has examined what role (if any) access to primary care has in preventing cognitive impairment. Mitigation of modifiable risk factors is a potential mechanism by which health care can prevent or delay cognitive impairment [3, 9]. Research on the relationship between cardiovascular health and dementia [10, 11] suggests that access to primary health care may mitigate age-related cognitive decline through effective management of vascular risk factors, such as hypertension and diabetes [12–14]. Other modifiable risk factors for dementia are also managed or encouraged by primary care physicians, including regular exercise, obesity and stopping smoking [9].

Therefore, we examine the association between the spatial availability of primary health care services in each REasons for Geographic And Racial Differences in Stroke (REGARDS) participant’s primary care service area (PCSA), individual access to regular medical care, and cognitive impairment (assessed through a well-validated cognitive screening instrument). PCSAs are a group of standardized primary care market areas developed with Medicare data that have been used to capture local access to care [15, 16]. Although access to care is commonly operationalized at the individual level as having health insurance, proximity to health care services at the geographic level is also a determining factor in accessing health care [17–20]. Because vascular risk factors including blood pressure, diabetes, and dyslipidemia increase risk of cognitive impairment, we hypothesize that residence in an area with more primary care physician supply will be associated with lower risk of cognitive impairment, in part through the better management of these conditions. We evaluate whether the management of individual vascular risk factors (hypertension, dyslipidemia, diabetes) accounts for any of the observed relationship between primary health care access and cognitive impairment.

While much research on health care and dementia has relied on clinical or patient samples [3, 21], such work excludes populations who have not been clinically diagnosed with dementia. The REGARDS study oversampled individuals living in the Stroke Belt states (e.g. Alabama, Louisiana, Mississippi, Tennessee), resulting in a sizable proportion of participants in rural areas and almost half the sample (42%) is African American. As a result, the study’s large community-dwelling sample includes those who are likely undercounted with respect to dementia diagnosis in existing research, and presents a unique opportunity to examine the role of primary care access for preventing cognitive impairment in some of the most vulnerable community-dwelling aging Americans.

## Methods

### Data

The REGARDS study is a national, population-based prospective cohort study examining stroke mortality [22]. Using mail and telephone contact methods, community-dwelling adults age 45 years or older were recruited from January 2003 to October 2007, stratified by age, race, sex, and geographic region. The cohort consists of 30,239 older adults (mean age 64 ± 9 years), with 16,934 from Stroke Belt states (Alabama, Arkansas, Georgia, Louisiana, Mississippi, North Carolina, South Carolina, and Tennessee) and 13,305 from the remaining 40 contiguous states and the District of Columbia. Because the largest stroke-related racial disparities are between black and white adults, REGARDS only enrolled those reporting race as either Black (42%) or White (58%).

A computer-assisted telephone interview was conducted to gather sociodemographic and health history information at baseline (2003-2007), followed by an in-home examination that collected physical measures (height, weight, blood pressure), blood and urine samples, and an inventory of current medications using pill bottle review. Full details of methods are available elsewhere [22]. Although the REGARDS study is ongoing, we focus on cross-sectional data from the baseline assessment, when the full suite of medication and physical measurement data were collected.

### Measures

#### Cognitive impairment

Cognitive impairment**,** was assessed using the Six-Item Screener (SIS) [23]. The SIS is a test of global cognitive function derived from the widely used Mini-Mental State Exam (MMSE), consisting of a 3-item word recall and 3-item temporal orientation (score range = 0-6). In community samples, a score < 5 indicates cognitive impairment [23–25], with 74.2% sensitivity and 80.2% specificity for clinically confirmed cognitive impairment no-dementia (CIND) and dementia [23]. The SIS has been validated against the MMSE, list learning tasks, and diagnoses of dementia and CIND in community-dwelling Black and White samples [23, 25]. We included REGARDS participants who had their first SIS by 2010.

#### Health care access

**Fig. 1:**
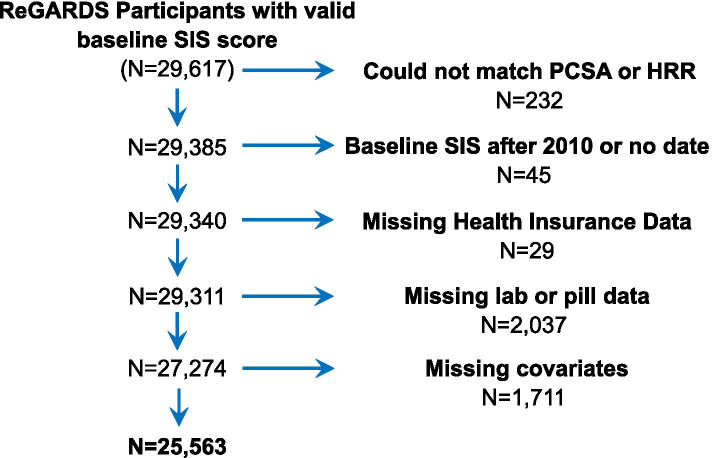
Participant exclusion flow diagram

**Fig. 2:**
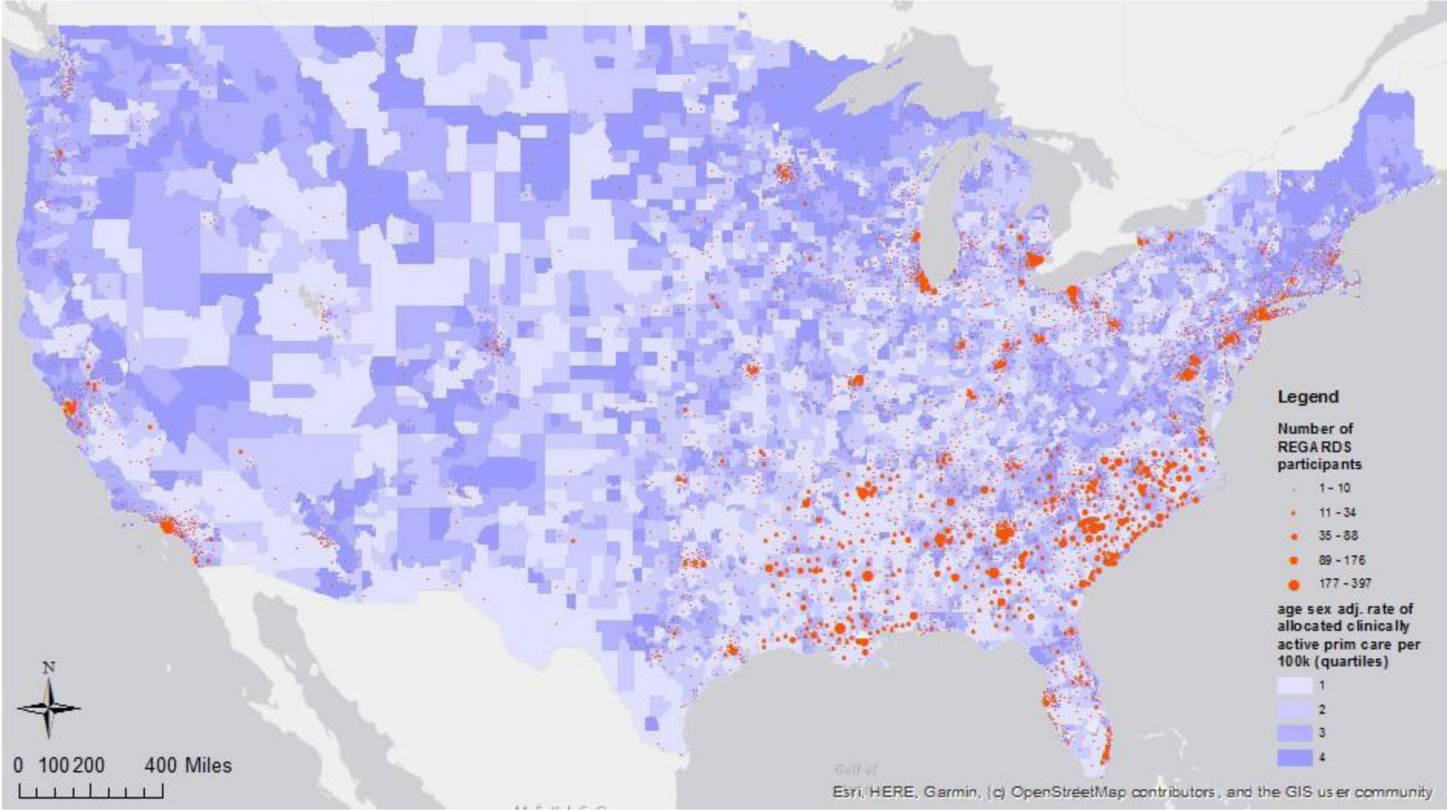
Distribution of REGARDS Participants by PCSA Primary Care Supply (2003-2007)

Participants were assigned to a primary care service area (PCSA) based on their residential address at the baseline visit [26]. Using geographic information systems (GIS), characteristics of health care resources in each participant’s PCSA were linked using the Spatial Join function in GIS software (ArcGIS, ESRI, Inc.) to the XY (latitude and longitude) coordinates of participants’ residential addresses. We included REGARDS participants who could be mapped to a PCSA (*n* = 29,340) (Fig. 1). We obtained data from Health Resources and Services Administration (HRSA) to characterize health care supply and utilization in each PCSA [27]. This publicly available HRSA dataset documents how medical resources are distributed and used in the United States. Using 2010 data, each PCSA in the US was ranked to derive measures of primary care supply and use, including a) national quartiles of the age and sex adjusted rate of clinically active primary care providers available per 100,000 census population; b) average annual rate of primary care visits per Medicare beneficiary; and c) the average annual rate of emergency department (ED) visits per Medicare beneficiary. Utilization of ED and primary care services were modeled as above or below the national median value. The distribution of REGARDS participants across these PCSAs is illustrated in Fig. 2. Individual access to care was operationalized as having a self-reported regular physician or clinic for medical care, and having health insurance. We excluded 29 Regards participants missing health insurance information (*n* = 29,311, Fig. 1).

#### Covariates

To evaluate the role of vascular health in the association between primary care and cognitive impairment, the management of diabetes, hypertension, and dyslipidemia were evaluated by data from physical measurements, blood/urine samples, and prescription medications. We excluded 2037 REGARDS participants missing lab values or pill audit data from the baseline visit (*n* = 27,274, Fig. 1).

Uncontrolled diabetes was defined as fasting blood glucose > = 126 mg/dL or non-fasting glucose > = 200 mg/dL. Controlled diabetes was defined as having a prescription medication for diabetes in the pill bottle audit, but having a normal blood sugar value (< 126 mg/dL fasted or < 200 mg/dL unfasted). No diabetes was defined as not having a diabetes medication, and having a normal blood sugar value.

Uncontrolled hypertension was defined as systolic blood pressure > =140 mmHg or diastolic blood pressure > =90 mmHg. Controlled hypertension was defined as having a hypertension medication in the pill bottle audit and having a normal blood pressure reading (systolic blood pressure < 140 mmHg, diastolic blood pressure < 90 mmHg). No hypertension was defined as not having a hypertension medication and having a normal blood pressure reading.

Uncontrolled dyslipidemia was defined as a lab measured HDL < =40 or LDL > =160. Controlled dyslipidemia was defined as having a prescription cholesterol medication and lab measured HDL > 40 and LDL < 160. No dyslipidemia was defined as not taking a cholesterol medication and having a normal lab measure (HDL > 40 or LDL < 160).

To account for potential selection of individuals at greater risk for cognitive impairment into PCSAs with less primary care, we adjusted for age (in years), gender (male, female), race (self-reported Black, White), education (<high school, high school graduate, some college, college degree or higher), marital status (married, not married (including separated, divorced, widowed and never married)), annual household income (<$20,000, $20,000-$34,999, $35,000-$74,999, $75,000+, or refused), cigarette smoking (number of packs smoked per year: none, >median (0.45) pack years, <median pack years), alcohol use (none (0 drinks per week), moderate (1-7 drinks/week for women; 1-14 drinks/week for men), or heavy (> 7 drinks/week for women; > 14 drinks/week for men)), exercise (none, 1-3 times per week, 4+ times per week). Body mass index (BMI, measured weight (kg)/height (m^2^)) was modeled as underweight (< 18.5), normal weight (18.5 to < 25), overweight (25 to < 30) and obese (> = 30). We excluded participants missing covariate data (*n* = 25,563, Fig. 1).

### Statistical analyses

The analytic sample included REGARDS participants with a baseline cognitive screening (SIS) test (*n* = 29,340). Participants were excluded from the analysis if they were missing demographic or behavioral measures (*n* = 1848) or physical measurements (blood pressure, LDL, HDL, glucose, BMI) from the baseline exam (*n* = 1929). The resulting analytic sample consisted of 25,563 participants (Fig. 1). We first described participant characteristics. Multivariable associations between cognitive impairment and PCSA characteristics were then modeled using a generalized estimating equation logistic regression with robust standard errors to account for clustering within PCSA. We conducted two sensitivity analyses to confirm the robustness of our findings to different definitions of vascular risk factor management. First, we defined vascular risk factors using lab test only definitions of diabetes, hypertension and dyslipidemia (not including medication information). Second, we modeled each of the vascular conditions separately. All analyses were conducted with STATA Version 15 software (College Station, TX), and statistical significance was assessed with a two-tailed alpha of 0.05. This study was approved by the University of Michigan Institutional Review Board (HUM00136943).

## Results

Table 1 presents the descriptive characteristics of the study sample. Of the 25,563 participants, 2145 (8.4%) were cognitively impaired at baseline and 23,418 (91.6%) were not. The median age at baseline was 64 (range 45-96 years). The majority of participants lived in PCSAs where the supply of primary care physicians was within the interquartile range of the nation, with slightly larger proportions of REGARDS participants living in areas at the second and third quartiles (31.0 and 27.7% respectively) compared to the nation as a whole. Most participants (56.5%) lived in PCSAs where primary care utilization was above the national median. Over half of participants (53.9%) lived in areas with ED utilization above the national median. The majority of participants reported a regular source of medical care (74.3%) and had health insurance (93.5%) (Table 1). There were 3381 distinct PCSAs in our sample with a range of 1 to 398 participants in each PCSA. There was an average of 7.6 participants per PCSA (standard deviation 20.0).

**Table 1:** Baseline Characteristics of Study Participants and their Primary Care Service Areas

	Participants with cognitive impairment (***n*** = 2145)	Participants without cognitive impairment (***n*** = 23,418)	Total	PCSAs (***n*** = 3381)
National quartile of age sex adjusted rate of allocated clinically active primary care supply in PCSA	N (%)	N (%)	N (%)	N (%)
Bottom 25%	525 (24.5)	5209 (22.2)	5734 (22.4)	807 (23.9)
Second lowest 25%	677 (31.6)	7238 (30.9)	7915 (31.0)	905 (26.8)
Second highest 25%	565 (26.3)	6528 (27.9)	7093 (27.7)	883 (26.1)
Top 25%	378 (17.6)	4443 (18.97)	4821 (18.9)	786 (23.2)
Primary Care Utilization in PCSA				
< US Median	869 (40.5)	10,246 (43.8)	11,115 (43.5)	1591 (47.1)
> US Median	1276 (59.5)	13,172 (56.3)	14,448 (56.5)	1790 (52.9)
ED Utilization in PCSA				
< US Median	974 (45.4)	10,822 (46.2)	11,796 (46.1)	1825 (54.0)
> US Median	1171 (54.6)	12,596 (53.8)	13,767 (53.9)	1556 (46.1)
Regular source of medical care				
Yes	1468 (68.4)	17,537 (74.9)	19,005 (74.3)	
No	454 (21.2)	4193 (17.9)	4647 (18.2)	
No Answer	223 (10.4)	1688 (7.2)	1911 (7.5)	
Insurance coverage				
Has health insurance	1999 (93.2)	21,891 (93.5)	23,890 (93.5)	
No health insurance	146 (6.8)	1527 (6.5)	1673 (6.5)	
Diabetes				
Managed	262 (12.2)	2181 (9.3)	2443 (9.6)	
Unmanaged	285 (13.3)	2387 (10.2)	2672 (10.5)	
No Diabetes	1598	18,850	20,448 (80.0)	
Hypertension				
Managed	934 (43.5)	9808 (41.9)	10,742 (42.0)	
Unmanaged	619 (28.9)	5063 (21.6)	5682 (22.2)	
No Hypertension	592 (27.6)	8547 (63.5)	9139 (35.8)	
Dyslipidemia				
Managed	495 (23.1)	5514 (23.6)	6009 (23.5)	
Unmanaged	734 (34.2)	7651 (32.7)	8385 (32.8)	
No Dyslipidemia	916 (42.7)	10,253 (43.7)	11,169 (43.7)	
Race				
White	883 (41.2)	14,378 (61.4)	15,261 (59.7)	
Black	1262 (58.8)	9040 (38.60)	10,302 (40.3)	
Sex				
Female	1047 (48.8)	13,113 (56.0)	14,160 (55.4)	
Male	1098 (51.2)	10,305 (44.0)	11,403 (44.6)	
Education				
Less than high school	532 (24.8)	2414 (10.3)	2946 (11.5)	
High school graduate	625 (29.1)	5956 (25.4)	6581 (25.7)	
Some college	503 (23.5)	6351 (27.1)	6854 (26.8)	
College degree or more	485 (22.6)	8697 (37.1)	9182 (35.9)	
Household Income				
< $20 K	608 (28.3)	3774 (16.1)	4382 (17.1)	
$20-34 K	627 (29.2)	5538 (23.7)	6165 (24.1)	
$35-74 K	467 (21.8)	7365 (31.5)	7832 (30.6)	
$75 K+	153 (7.1)	4079 (17.4)	4232 (16.6)	
Refused to Answer	290 (13.5)	2662 (11.4)	2952 (11.5)	
Relationship status				
Not currently married	1004 (46.8)	9247 (39.5)	10,251 (40.1)	
Married	1141 (53.2)	14,171 (60.5)	15,312 (59.9)	
Pack years of cigarette smoking				
Zero pack years	970 (45.2)	11,139 (47.6)	12,109 (47.4)	
Below the median (0.45) pack years	549 (25.6)	6142 (26.2)	6691 (26.2)	
Above the median (0.45) pack years	626 (29.2)	6137 (26.2)	6763 (26.5)	
Alcohol drinking frequency				
Never	1498 (69.8)	14,340 (61.2)	15,838 (62.0)	
Moderate alcohol use	562 (26.2)	8144 (34.8)	8706 (34.1)	
Heavy alcohol use	85 (3.96)	934 (3.99)	1019 (4.0)	
Exercise				
No exercise	830 (38.7)	7711 (32.9)	8541 (33.4)	
Exercise 1-3 times per week	643 (29.98)	8735 (37.3)	9378 (36.7)	
Exercise 4+ times per week	672 (31.3)	6972 (29.8)	7644 (29.9)	
Body Mass Index				
Underweight	34 (1.6)	227 (0.97)	261 (1.0)	
Normal weight	521 (24.3)	5543 (23.7)	6064 (23.7)	
Overweight	817 (38.1)	8701 (37.2)	9518 (37.2)	
Obese	773 (36.0)	8947 (38.2)	9720 (38.0)	

*PCSA* Primary Care Service Area, *ED* Emergency Department

In un-adjusted multivariable generalized estimating equation logistic models of individual and area-level access to primary care, compared to those living in a PCSA in the highest primary supply quartile, those living in a PCSA in the lowest quartile of primary care supply had 32% higher odds of cognitive impairment (OR 1.32; CI 1.12, 1.56), and those living in the second lowest quartile of supply had 18% higher odds of cognitive impairment (OR 1.18; CI 1.01, 1.38). Living in a PCSA with ED utilization over the US median level was not associated with higher odds of cognitive impairment (OR 1.02; CI 0.92, 1.13), but PCSA primary care utilization over the median level was associated with 14% higher odds of cognitive impairment (OR 1.14; CI 1.03, 1.27). Not having a regular source of medical care was associated with 29% higher odds of cognitive impairment (OR 1.29; CI 1.14, 1.45), but not having health insurance was not associated with higher odds of cognitive impairment (OR 0.96; CI 0.80, 1.16).

**Fig. 3:**
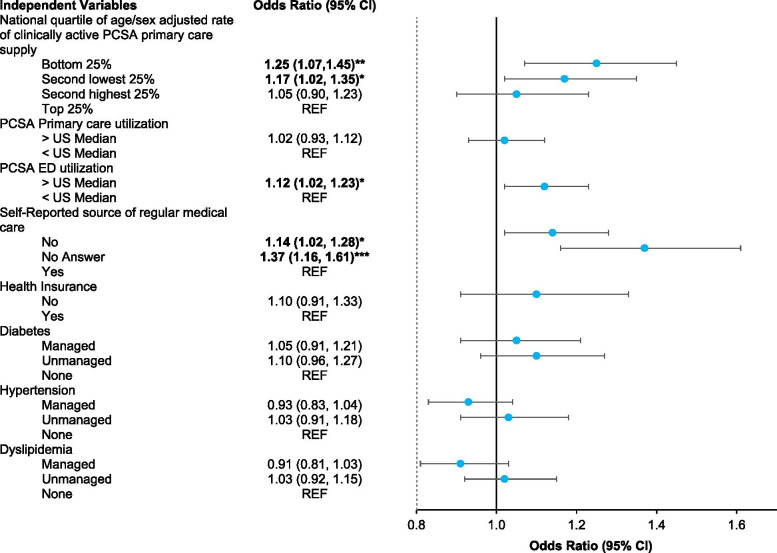
Results from Generalized Estimating Equation Models of Cognitive Impairment by Individual and Primary Care Service Area Characteristics. Models adjust for age, gender, race, education, income, smoking, alcohol use, exercise, body mass index. PCSA = Primary Care Service Area; CI=Confidence Interval; ED = Emergency Department. Boldface indicates statistical significance (*p < 0.05; **p < 0.01; ***p < 0.001)

Figure 3 presents the results (odds ratios (OR) and 95% confidence intervals (CI)) from the fully adjusted analyses. Lower primary care supply was associated with higher odds of cognitive impairment, net of individual sociodemographic and health risk factors. Compared to those living in a PCSA in the highest quartile of primary care supply, living in a PCSA in the lowest quartile of supply was associated with 25% higher odds of cognitive impairment (OR 1.25; CI 1.07, 1.45) and living in a PCSA in the second lowest quartile was associated with 17% higher odds of cognitive impairment (OR 1.17; CI 1.02, 1.35). Living in a PCSA with ED utilization over the median level in the US was associated with 12% higher odds of cognitive impairment (OR 1.12; CI 1.02, 1.23), but there was no association with PCSA primary care utilization over the median level. Not having a regular source of medical care was associated with 14% higher odds of cognitive impairment (OR 1.14; CI 1.02, 1.28).

Although odds ratios were in the expected directions, baseline status of managed/unmanaged diabetes, managed/unmanaged hypertension, and managed/unmanaged dyslipidemia were not statistically significantly associated with cognitive impairment at baseline, nor did they change the effect estimates of the PCSA variables when added in the models sequentially (Supplemental Table 1). A sensitivity analysis) using lab only definitions of diabetes, hypertension and dyslipidemia did not yield meaningfully different results, nor did modelling these vascular conditions separately (Supplemental Table 2).

## Discussion

To our knowledge, access to primary care has not been studied in relation to cognitive impairment despite its important role in the prevention of several known vascular risk factors. Using a large nationwide study of community-dwelling aging Americans, we found that beyond an individual’s health insurance status, education, and income, living in an area with more primary care physician supply was associated with better cognitive health in older Americans. Specifically, residence in a PCSA with more primary care physicians was associated with 25% lower odds of cognitive impairment. Beyond the availability of primary care supply, we found that having a regular source of primary care was associated with lower odds of cognitive impairment. Our results add to the existing body of research suggesting that having a regular primary care provider may be particularly important for building clinician-patient-family relationships, coordinating the management of risk factors, and understanding and recognizing patients’ cognitive changes over time [21].

Area level primary care utilization was not associated with cognitive impairment, but higher rate of ED utilization at the PCSA level was associated with increased odds of cognitive impairment. Higher use of emergency departments in the community may reflect issues with care coordination, lack of an annual checkup, or low perceived access to care [28]. Indeed, we found that there is a potential benefit to reporting a regular source of health care over and above area level primary physician supply and utilization. This is consistent with research on older adults with dementia which found that low continuity of care was associated with higher utilization of health care [21].

We did not find associations between vascular risk factor management (management of hypertension, dyslipidemia, and diabetes) and cognitive impairment. However, we only had a one-time measurement for vascular risk factors and midlife health has lasting effects on the risk of cognitive impairment [29, 30]. Primary care access may have a longer acting effect that is not captured in our measurement. There are also a host of other preventive measures implemented in the primary care setting that that might be operating in the results we see, including targeting regular physical activity, healthy diet and lifelong learning/cognitive training [9].

Existing research has focused less on the role of ambulatory and primary care for preventing cognitive impairment, and instead has examined the primary care setting for its role in detecting, diagnosing, and treating cognitive impairment and dementia [8, 21, 31, 32]. Medicare coverage of the annual wellness visit that includes the assessment of cognitive function has not shown to be effective in detecting cognitive impairment or ADRD, at least in early assessments [32]. It is therefore possible that primary care could have a more effective role in preventing the onset of cognitive impairment rather than its detection. *The Lancet Commission on Dementia Prevention, Intervention, and Care* concluded that 35% of dementia cases could be prevented by modifying nine risk factors: low education, midlife hearing loss, obesity and hypertension, late-life depression, smoking, physical inactivity, diabetes, and social isolation [33]. The findings from a recent update added excessive alcohol consumption, head injury, and air pollution to this list [34]. It is estimated that modifying these 12 risk factors, all of which are managed in the primary care setting, might prevent or delay up to 40% of dementias [34]. A focus on multi-domain interventions, targeting more than one risk factor, may be more effectively delivered in the primary care setting [7].

While the strengths of this work include the addition of linked health care resource data to screening measures of cognitive function in the REGARDS cohort, it is not known whether the presence of health care in one’s community is actually related to health care use. However, because cognitive impairment was assessed through a screening instrument, this study avoids the limitations of previous work with patient samples that are contingent on a diagnosis of cognitively impaired no dementia (CIND) or dementia [4, 21]. Thus, we were able to examine the potential role of health care for cognitive function even among those whose cognitive status has not been assessed/detected by a physician or before symptoms reach a threshold for a dementia diagnosis, which broadens the generalizability of our findings. While we did see racial disparities in cognitive impairment, we did not find racial inequities in the relationship between primary care and cognitive impairment (Supplemental Table 3). Further work should delve deeper into racial differences in access to care and perceptions of access [35]. This analysis was cross-sectional and management of risk factors was assessed at a single time point. Given the importance of exposure timing and different cognitive trajectories, future studies should incorporate longitudinal measures of vascular health and other risk factors such as impaired hearing to better understand the mechanism of primary care. Finally, given the cross-sectional nature of this analysis, we cannot rule out reverse causation. Although it is possible that cognitive impairment influences where a person lives, this is unlikely to explain the association we see with PCSA primary care supply and cognitive impairment because PCSA primary care supply is determined by different factors than the factors that select people into different neighborhoods (e.g, income) [36].

## Conclusions

The finding that the availability of primary health care and having a regular source of care were associated with reduced risk of cognitive impairment, net of individual risk factors and characteristics that could be a source of selection bias, is novel and important for dementia planning and prevention. Participants in our study were not clinically diagnosed with CIND or dementia, raising the importance of this finding. This paper is an important step in understanding the role of the healthcare system for mitigating and preventing cognitive impairment and ADRD.

## Supplementary Information

**Additional file 1.**

**Additional file 2.**

**Additional file 3.**

## Data Availability

REGARDS data are available with a reasonable proposal. Contact REGARDSAdmin@uab.edu. Data on PCSA level primary care supply and utilization are publically available from Health Resources and Services Administration and can be downloaded from their website [27].

## Abbreviations

ADRD: Alzheimer’s disease and related dementias
REGARDS: Reasons for Geographic and Racial Differences in Stroke
SIS: Six item screener
ED: Emergency department
PCSA: Primary care service area
MMSE: Mini-mental state exam
CIND: Cognitive impairment no dementia
GIS: Geographic information system
